# “Getting Engaged”

**DOI:** 10.1007/s11606-014-2948-0

**Published:** 2014-07-08

**Authors:** Jean Slutsky, Sue Sheridan, Joe Selby

**Affiliations:** Patient-Centered Outcomes Research Institute (PCORI), 1828 L Street, NW 9th Floor, Washington, DC 20036 USA

The paper by Concannon et al. in this issue of JGIM raises several thoughtful themes about the relationship between researchers who study pragmatic health questions and those individuals—patients, clinicians, institutions—who use the study findings to make decisions about their own health care or that of patients or populations.^1^ As pointed out by the authors, meaningful engagement of stakeholders in planning, conducting, and disseminating research is thought to help make research findings more “useful” and “used.” Engagement is intended to help in making research questions more relevant and to ensure that the research is conducted and the findings interpreted with stakeholders’ viewpoints at the table. Engagement may also serve to enhance recruitment of study participants, and ultimately should lead to greater uptake of research findings when the study is finished.

However, methods for establishing and maintain meaningful engagement are not well-defined and may be challenging. The frameworks developed by Concannon et al. for categorizing types of stakeholders and stages of research are useful starting points for developing and evaluating engagement strategies. The descriptive information in their report from published studies raises two concerns worth mentioning. First, engagement of patients was mentioned more frequently than engagement of clinicians, and much more frequently than engagement of payers and purchasers. While this could simply be an artifact due to the authors’ mentioning patient engagement more often because it seemed more novel, we suspect that it is more representative of reality than not.

The Patient-Centered Outcomes Research Institute (PCORI) has established itself on a foundation of ongoing, meaningful engagement of all participants in the health care system throughout the research cycle. Its Methodology Standards^2^ recommend engagement and its funding announcements require it.

Because patients are the central focus of health care delivery, it is logical that their wants, values, and challenges are highly prioritized in studying health care choices. Indeed, this principle is recognized around the world and across multiple fields of clinical research, from international efforts of the World Health Organization^3^ to drug development research at the Food and Drug Administration in the US.^4^

However, if other relevant stakeholder groups are not also involved, we do not expect that the research would be sufficiently representative of the stakeholders’ information needs, environment, or priorities, therefore jeopardizing buy in, practice changes and improved outcomes.

We would also emphasize our experience that the dialogue of engagement should be multi-directional among all stakeholder groups, rather than a series of bi-directional discussions between each group and researchers. Placing patient interests at the center, this dialogue allows diverse groups to gain insight into the concerns and information needs of others, and to shape research questions that are mutually agreeable.

A second concern is the concentration of engagement activities in the earlier stages of the research process, rather than in the later stages of interpreting findings and disseminating results. This is concerning, since the central reason for engagement is to enhance dissemination and uptake of relevant research findings. In some of the studies reviewed, dissemination may not have been warranted, and in others, researchers may simply not have attempted to disseminate findings or chosen not to report on these efforts. Nevertheless, this finding is also likely to reflect reality, and emphasizes the need to engage with stakeholder organizations, as well as with individuals, to further dissemination.

An area of engagement emphasized by PCORI but not included in the authors’ framework is the review of research funding applications—not surprising because they focus on engagement within research projects. Review of research applications has traditionally been conducted by “peers,” by “peers,” i.e., other researchers. PCORI has deliberately introduced a “merit” review process to emphasize evaluation of proposed research from several perspectives: that of the scientist, but also those of patients and other relevant stakeholders such as clinicians, businesses, private and public payers, the life science industry, hospitals and health systems, policymakers and training institutions, etc. Including non-scientific reviewers in a traditionally scientific discussion has presented challenges for all participants, but has also been called refreshing and rewarding in post-review interviews and in a recent blog by a patient reviewer.^5^ Despite the positive feedback from merit reviewers, the empirical evidence must be gathered to support the anecdotal evidence that engaging non-scientists in peer review results in more relevant studies. Data from the first round of merit review suggest that all reviewers are influenced by other perspectives during these face-to-face discussions and that scores tend to converge on a somewhat different set of top-scoring applications after discussion.^6^ Further evaluation (as described below) will determine if these reviews lead to better, more useful studies.

The authors recognize the need for a “science of engagement,” and have contributed to launching this inquiry with frameworks and data collection tools. The dramatic changes in the research process required by PCORI call for careful study and evaluation of stakeholder engagement. PCORI will routinely monitor data on a number of outcomes that could reflect effects of engagement.^7^ Outcomes include the usefulness of the funded research questions and of completed studies, assessed through pre-specified usefulness criteria and stakeholder surveys; familiarity with and trust in PCORI-funded research by patients and other stakeholders; participation rates in research projects among eligible subjects and the frequency of success in dissemination and implementation. PCORI will also support research in this area by others through our research methods funding announcement.

There is also a need to understand the mechanisms by which engagement exerts beneficial impacts, if any. Of particular interest to PCORI is the frequency with which stakeholders influence the choice of the research questions, comparators, or outcomes; and whether the partnerships of researchers, patients, clinicians and other stakeholders develop studies or recruitment practices that are more attractive to potential participants. Finally, we are curious to explore whether the participation and endorsement of patients in research may influence institutional review boards to weigh the potential benefits of these the research more favorably in relationship to their typically low risk.

Perhaps most challenging, as suggested by the Concannon et al. review, is achieving and sustaining true engagement of stakeholders in the fabric of the research study, from beginning to end. Most major funding organizations do not currently require this type of prolonged and relatively intense engagement. PCORI has recognized these challenges and put several activities in motion to assist in bridging possible gaps between researchers and stakeholders. PCORI offers training for both researchers and stakeholders, including a guide for engaging patients and families in research; sample engagement plans; extensive merit review training and post-merit review surveys to better understand the challenges of diverse merit review panels; and small competitive awards to allow researchers and stakeholders to build relationships before an application is developed and submitted.

Identifying the best practices in engagement, including the amounts, timing and venues, remains a work in progress. A key question requiring further study is that of compensation for stakeholder participants. For some stakeholders, this activity may be valued by their employer. For others, the appropriate level of compensation is not settled, although it certainly differs by the intensity of engagement. For some vulnerable patient populations, monetary compensation could in theory create a conflict for patients on assistance programs. For some non-patient stakeholders, potential conflicts of interest will need to be addressed.

In conclusion, it is encouraging that the Concannon et al. review was able to document engagement in many aspects of research. PCORI remains committed to promoting intensive engagement of all appropriate stakeholders. The gaps, challenges and unknowns provide a roadmap for researchers and funding organizations to follow to make sure that engagement becomes both robust and efficient.
